# Equations of motion for weakly compressible point vortices

**DOI:** 10.1098/rsta.2021.0052

**Published:** 2022-06-27

**Authors:** Stefan G. Llewellyn Smith, T. Chu, Z. Hu

**Affiliations:** ^1^ Department of Mechanical and Aerospace Engineering, Jacobs School of Engineering, University of California San Diego, 9500 Gilman Drive, La Jolla, CA 92093-0411, USA; ^2^ Scripps Institution of Oceanography, University of California San Diego, 9500 Gilman Drive, La Jolla, CA 92093-0209, USA; ^3^ Department of Mechanical Engineering, Stanford University, Stanford, CA 94305, USA

**Keywords:** Mach number, Rayleigh–Jansen expansion, matched asymptotic expansions, point vortices

## Abstract

Equations of motion for compressible point vortices in the plane are obtained in the limit of small Mach number, *M*, using a Rayleigh–Jansen expansion and the method of Matched Asymptotic Expansions. The solution in the region between vortices is matched to solutions around each vortex core. The motion of the vortices is modified over long time scales $O(M^{2}\log M)$ and $O(M^{2})$. Examples are given for co-rotating and co-propagating vortex pairs. The former show a correction to the rotation rate and, in general, to the centre and radius of rotation, while the latter recover the known result that the steady propagation velocity is unchanged. For unsteady configurations, the vortex solution matches to a far field in which acoustic waves are radiated.

This article is part of the theme issue ‘Mathematical problems in physical fluid dynamics (part 2)’.

## 1. Introduction

Vorticity is a key aspect of fluid mechanics, in particular in high-Reynolds-number and turbulent flows. Many flows are dominated by intense vortices, and efforts to obtain reduced systems and equations have led to the development of models with singular vorticity distributions. Of these, point vortices in the plane are the simplest example, with the vortices’ motion being governed by a set of ODEs. A discussion of the justification for the equations of motion of point vortices and generalizations is given in [1], including a review of the momentum argument set out by [2].

The majority of the work to date on point vortices has been for plane incompressible flows [2]. Attempting to extend the notion of a point vortex to plane compressible flows is a daunting task in the general case, but for the case of low-Mach number flows, a Rayleigh–Jansen expansion in Mach number provides one approach. With the Mach number used in the expansion defined by the velocities induced by the vortices’ motion and the speed of sound, the O(1) incompressible velocity field increases so as to become supersonic near the location of a point vortex. One needs to consider further physics near the vortex location, i.e. the vortices have a small core region. Barsony–Nagy, Er-El & Yungster (hereafter BNEEY) [3] showed how to obtain steady point vortex configurations in this manner, relating the core behaviour to a solution obtained by Taylor [4].

Since then, there have been a few similar studies. These have examined the translating vortex pair [5–7], for which it was found in [7] (hereafter L06) that the speed of propagation was unchanged at $O(M^{2}$), and the von Kármán vortex street [8], for which the speed of propagation for both staggered and unstaggered streets can either increase or decrease depending on parameters of the flow. (There have also been works on steady weakly compressible hollow vortices, as in [9–11], but these do not consider point vortices.)

As pointed out by [5], the existence of a family of continuous shock-free transonic compressible flows with embedded vortices is of intrinsic interest, given that similar flows for transonic aerofoils do not persist under small perturbations. Our goal is to extend the work on weakly compressible point vortices to the unsteady case. We extend the approach of BNEEY to obtain equations of motion for the positions of the vortices up to $O(M^{2}$). Our approach is based on conservation of momentum, which has been used for incompressible constant-density flows and which we now review (see [1]). We compute the rate of change of momentum inside a moving closed contour C from Newton’s Second Law in complex notation,

1.1 $$\frac{\text{d}P}{\text{d}t}=\text{i}\oint_{C}p\;\text{d}z-\frac{\text{i}}{2}\oint_{C}\rho \bar{w}[(\bar{w}-\bar{W})\;\text{d}\bar{z}-(w-W)\;\text{d}z],$$

where the contour C is described in the positive sense, the complex momentum inside C is given by the area integral $P=\int_{S}\rho \bar{w}\;\text{d}S$, the complex position and velocity are z=x+iy and w=u−iv respectively, and the velocity of C is given by W=U−iV. Using Bernoulli’s equation, substituting local expansions for the variables into (1.1), and taking the limit as the contour shrinks down to the vortex, gives ${\bar{\zeta }}_{t}=W=\tilde{w}$, since the contour moves with the vortex. Here, $\tilde{w}$ is the desingularized velocity at the vortex: physically, a point vortex moves with the local desingularized flow.

In this incompressible argument, point vortices have no internal structures, but for the compressible case, we will need to consider the flow in the vortex cores on scales of O(M) smaller than the distance between vortices. In addition, at large distances of $O(M^{-1})$ from the vortical region, as pointed out by L06, the Rayleigh–Jansen expansion will become disordered, indicating the presence of a wavelike far field. This feature was already present in previous work on sound generation by vortical flows in aeroacoustics using matched asymptotic expansions (MAE; see [12,13] for an overview).

**Figure 1.**
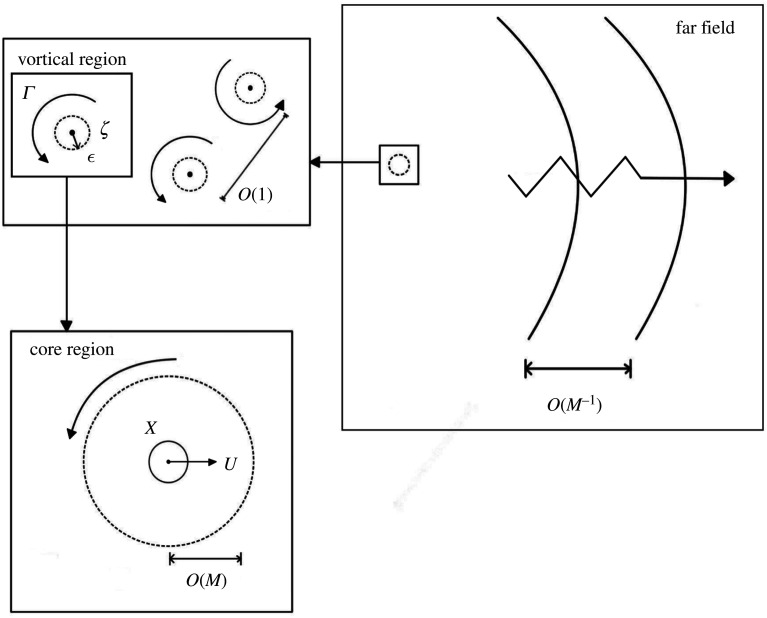
Schematic illustrating the different regions of the flow.

Terms in the Rayleigh–Jansen expansion will evolve on slow time scales to allow the position of vortices to change. Figure 1 illustrates the different regions of the flow and gives some notation. In §2, we present the governing equations for the vortical region, and then examine the core region to understand the nature of the expansion. In §3, we obtain the equations of motion for a vortex to $O(M^{2})$ by solving at successive orders in the Rayleigh–Jansen expansion, and then discuss the form of the global solution. We examine the case of two compressible vortices in §4. We discuss the far field in §5 and relate it to previous work. Finally, §6 concludes the paper. Electronic supplementary material includes details of algebra, as well as an account of the formal matching near the vortex cores.

## 2. Problem formulation

We consider irrotational adiabatic compressible flow in the plane. The adiabatic relation between pressure, $p^{*}$, and density, $\rho^{*}$, takes the form $p^{*}/p_{0}^{*}={\left(\rho^{*}/\rho_{0}^{*}\right)}^{\gamma }$, where γ is the constant ratio of specific heats, and $p_{0}^{*}$ and $\rho_{0}^{*}$ are reference values for pressure and density, taken to be the values at infinity where the flow is at rest. The momentum equation can be transformed into the unsteady Bernoulli equation,

2.1 $$\frac{\partial \phi^{*}}{\partial t}+\frac{1}{2}|\nabla \phi^{*}{|}^{2}+\int \frac{\text{d}p^{*}}{\rho^{*}}=\frac{\partial \phi^{*}}{\partial t}+\frac{1}{2}|\nabla \phi^{*}{|}^{2}+\frac{c^{2}}{\gamma -1}=\frac{c_{0}^{2}}{\gamma -1},$$

where $\phi^{*}$ is the velocity potential and the speed of sound (squared) is given by $c^{2}=\text{d}p^{*}/\text{d}\rho^{*}{|}_{s^{*}}=\gamma p^{*}/\rho^{*}$ with constant value $c_{0}^{2}=\gamma p_{0}^{*}/\rho_{0}^{*}$ at infinity. It is convenient to combine the above equation and the continuity equation into a single equation for the velocity potential, the Blokhintsev equation [7,14].

We non-dimensionalize using a length characteristic of the distance between vortices *L*, a typical velocity *V* induced by one vortex on another, the resulting time scale L/V, as well as the value of density at large distances, $\rho_{0}$ and the dynamic pressure scale $\rho_{0}V^{2}$. Then the Mach number is $M=V/c_{0}$, and the Blokhintsev equation becomes, dropping the stars and using the summation convention with subscripts running from 1 to 2,

2.2 $$\nabla^{2}\phi =M^{2}\left\{(\gamma -1)\left(\frac{\partial \phi }{\partial t}+\frac{1}{2}|\nabla \phi {|}^{2}\right)\nabla^{2}\phi +\frac{\partial^{2}\phi }{\partial t^{2}}+2\frac{\partial \phi }{\partial x_{i}}\frac{\partial^{2}\phi }{\partial x_{i}\partial t}+\frac{\partial \phi }{\partial x_{i}}\frac{\partial \phi }{\partial x_{j}}\frac{\partial^{2}\phi }{\partial x_{i}\partial x_{j}}\right\},$$

along with

2.3 $$p=\frac{1}{\gamma M^{2}}{\left[1-(\gamma -1)M^{2}\left(\frac{\partial \phi }{\partial t}+\frac{1}{2}|\nabla \phi {|}^{2}\right)\right]}^{\gamma /(\gamma -1)}$$

and

2.4 $$\rho ={\left[1-(\gamma -1)M^{2}\left(\frac{\partial \phi }{\partial t}+\frac{1}{2}|\nabla \phi {|}^{2}\right)\right]}^{1/(\gamma -1)}.$$

The above equations are valid in the region of length scale *L* between vortices, which we call the vortex region. They break down near the vortex cores, as pointed out by BNEEY and also examined by L06. To understand the flow behaviour in a vortex core and its impact on the subsequent matching process, we work in a reference frame co-moving with the vortex, so that for a vortex at location X moving with velocity U, one has

2.5 $$x=\hat{x}+X,\;u=\hat{u}+U,\;t=\hat{t}\;\mathrm{and}\;\phi =\hat{\Phi }+U\cdot \hat{x},$$

where $\hat{x}$ and $\hat{u}$ are the position and velocity with respect to the vortex core in the moving frame, respectively. Here, $\hat{t}$ is used to emphasize that partial time- and space-derivatives in the core frame are taken with constant $\hat{x}$ and $\hat{t}$, respectively. The velocity potential in the core frame is $\hat{\Phi }$. Following previous authors, we now define an appropriately scaled variable in the core region using $\hat{x}=Ms$, so that the radial coordinate measured from the vortex core is r=Ms. In terms of these variables, the Blokhintsev equation becomes

2.6 $$\begin{array}{rl} & \left[1-(\gamma -1)\left(M^{2}\frac{\partial \hat{\Phi }}{\partial \hat{t}}+\frac{1}{2}\frac{\partial \hat{\Phi }}{\partial s_{j}}\frac{\partial \hat{\Phi }}{\partial s_{j}}+M^{3}{\dot{U}}_{j}s_{j}-\frac{1}{2}M^{2}U_{j}U_{j}\right)\right]\frac{\partial^{2}\hat{\Phi }}{\partial s_{i}^{2}}\\ & \;\;=M^{4}\frac{\partial^{2}\hat{\Phi }}{\partial {\hat{t}}^{2}}+2M^{2}\frac{\partial \hat{\Phi }}{\partial s_{i}}\frac{\partial^{2}\hat{\Phi }}{\partial s_{i}\partial \hat{t}}+M^{6}\frac{\partial \hat{\Phi }}{\partial s_{i}}\frac{\partial \hat{\Phi }}{\partial s_{j}}\frac{\partial^{2}\hat{\Phi }}{\partial s_{i}\partial s_{j}}\\ & \;\;+M^{5}{\ddot{U}}_{j}s_{j}-M^{4}U_{j}U_{j}+M^{3}{\dot{U}}_{j}\frac{\partial \hat{\Phi }}{\partial s_{j}}, \end{array}$$

where dots above $U_{j}$ indicate time derivatives.

The Rayleigh–Jansen expansion is an expansion in small Mach number. Before writing it down in §3, we consider the momentum equation in complex form, as in [1], taken over the small circle with radius *e*. We take e≫M, as we are interested in the momentum balance over circles that are asymptotically small with respect to the region between vortices but much larger than the vortex cores, i.e. *e* is an intermediate variable in the terminology of MAE. This means that one can use either the core solution or the vortex solution when evaluating the right-hand side of (1.1), since the terms on the right-hand side are all contour integrals evaluated at radius *e*. The left-hand side, however, is a surface integral that must be calculated in the inner variable. To leading order in *M*, we have $\hat{\Phi }=\kappa \theta$ where κ=Γ/(2π) is the scaled circulation and θ is the polar angle, so that in vectorial form, the momentum is

2.7 $$\begin{array}{rl} & \;\oint \int_{s_{v}}^{e/M}\hat{\rho }(M^{-1}\hat{\nabla }\Phi )M^{2}s\;\text{d}s\;\text{d}\theta \approx M^{2}\oint \int_{s_{v}}^{e/M}{\left[1-(\gamma -1)\frac{\kappa^{2}}{2s^{2}}\right]}^{1/(\gamma -1)}\left[\frac{\kappa }{Ms}t+U\right]s\;\text{d}s\;\text{d}\theta , \end{array}$$

where t is the unit vector tangential to the circle. The lower limit $s_{v}$ is the smallest value of *s* for which the pressure and density in (2.3) and (2.4) are positive, and is obtained from the condition $2{s_{v}}^{2}=(\gamma -1)\kappa^{2}$. We see that the term in t cancels by symmetry. In complex form, we then obtain

2.8 $$P=\pi \bar{W}\left[e^{2}+\kappa^{2}M^{2}\log M-\kappa^{2}M^{2}\log e+M^{2}C+O\left(\frac{M^{4}}{e^{2}}\right)\right],$$

for e≫M, where

2.9 $$\begin{array}{rl} C & \;=2\int_{s_{v}}^{\infty }\left\{{\left[1-(\gamma -1)\frac{\kappa^{2}}{2s^{2}}\right]}^{\beta }-1+\frac{\kappa^{2}}{2s^{2}}\right\}s\;\text{d}s-{s_{v}}^{2}+\kappa^{2}\log s_{v} \end{array}$$

2.10 $$\begin{array}{rl} & \;=2\beta [\psi (\beta )-\psi (1)-1]+2-{s_{v}}^{2}+\kappa^{2}\log s_{v}, \end{array}$$

where ψ(z) is the digamma function and we write $\beta ={\left(\gamma -1\right)}^{-1}$ for brevity. We see that (2.8) contains a term of $O(M^{2}\log M)$ if the flow is unsteady. This means that such a term must exist in appropriate time-derivatives of the Rayleigh–Jansen expansion, either as a term in the expansion or as a result of slow time variation.

## 3. Derivation of the equation of motion for a vortex

### (a) Rayleigh–Jansen expansion of the global solution in the vortical region and time dependence

Motivated by the discussion above, we consider a modified version of the Rayleigh–Jansen expansion in the form

3.1 $$\phi (t,z,\bar{z})=\phi_{0}(t,z)+M^{2}\log M\phi_{1}(t,z)+M^{2}\phi_{2}(t,z,\bar{z})+\cdots ,$$

where the arguments of the two first terms reflect the fact that they correspond to incompressible flow in the vortical region, since from (2.2) we have $\nabla^{2}\phi_{0}=\nabla^{2}\phi_{1}=0$. The governing equation for $\phi_{2}$,

3.2 $$\nabla^{2}\phi_{2}=\frac{\partial^{2}\phi_{0}}{\partial t^{2}}+2\frac{\partial \phi_{0}}{\partial x_{i}}\frac{\partial^{2}\phi_{0}}{\partial x_{i}\partial t}+\frac{\partial \phi_{0}}{\partial x_{i}}\frac{\partial \phi_{0}}{\partial x_{j}}\frac{\partial^{2}\phi_{0}}{\partial x_{i}\partial x_{j}},$$

does not contain γ.

Define a complex potential $F_{0}=\phi_{0}+\text{i}\psi_{0}$, since the flow at leading order is incompressible and irrotational. Similarly, there is a complex potential $F_{1}=\phi_{1}+\text{i}\psi_{1}$. These potentials are harmonic functions that decay far from the vortex. Since $F_{0}$ has logarithmic singularities, $F_{1}$ cannot have singularities of higher order, while logarithmic singularities in $F_{1}$ are disallowed by requiring the vorticity to be entirely at O(1). Hence, as an analytic function bounded at infinity with no singularities, $F_{1}$ is a constant that can be taken to be 0 without loss of generality. This means that the Rayleigh–Jansen expansion does not in fact have a term at $O(M^{2}\log M)$. The term entering the matching from (2.8) must therefore come from taking the $O(M^{2}\log M)$ time dependence into account appropriately, as is done in (3.6) below.

Using $\phi_{0}=(F_{0}+\bar{F_{0}})/2$, (3.2) becomes

3.3 $$\nabla^{2}\phi_{2}=2\frac{\partial^{2}F_{2}}{\partial z\partial \bar{z}}+\text{c.c.}=\frac{1}{2}(F_{0tt}+2F_{0z}\bar{F_{0zt}}+F_{0zz}\bar{F_{0z}^{2}}+\text{c.c.}),$$

where c.c. stands for complex conjugate and we have defined a function $F_{2}(t,z,\bar{z})$ such that $\phi_{2}=(F_{2}+{\bar{F}}_{2})/2$. Note that, because the flow at $O(M^{2})$ is no longer incompressible, there is no streamfunction corresponding to $\phi_{2}$, so we call $F_{2}$ a potential but not a complex potential. Only the real part of $F_{2}$ matters, and the complex velocity $w_{2}=u_{2}-\text{i}v_{2}$ is given by $w_{2}=\partial_{z}(F_{2}+{\bar{F}}_{2})$. We can integrate (3.3) and obtain a particular solution for $\phi_{2}$ as the real part of

3.4 $$F_{2}(z,\bar{z})=\frac{1}{4}(z-Z_{0})\bar{J(z)}+\frac{1}{2}F_{0t}(z)\bar{F_{0}(z)}+\frac{1}{4}w_{0}(z)\bar{I(z)}+G(z),$$

where $Z_{0}$ is a time-dependent centre of vorticity that can be picked to simplify the analysis for specific cases. The functions I(z) and J(z) are defined globally by

3.5 $$I(z)=\int_{z_{I}}^{z}w_{0}^{2}\;\text{d}z\;\mathrm{and}\;J(z)=\int_{z_{J}}^{z}F_{0tt}\;\text{d}z.$$

The integration limits $z_{I}$ and $z_{J}$ will also be picked depending on the global nature of the flow. The full $O(M^{2})$ potential (3.4) is composed of an inhomogeneous part and a homogeneous part, G(z). The function G(z) is made up of homogeneous solutions of the Poisson equation (3.2), i.e. solutions of Laplace’s equation that can be written as functions of *z*. They are used to enforce single-valuedness of the velocity field, appropriate behaviour near the vortices and boundary conditions.

It turns out that the location of the vortex, considered as an expansion in Mach number, is not uniquely defined. We can remove this ambiguity by requiring that the location not be expanded in Mach number (this is reminiscent of slaving principles as in [15]). However, we need to allow the position to evolve in time at higher order in *M* to allow matching of the terms in (2.8). This leads to the use of multiple time scales. Given the form of (2.8), we consider all variables to be functions of $t_{0}=t$, $t_{1}=tM^{2}\log M$ and $t_{2}=tM^{2}$ (and possibly further time scales). We define

3.6 $$\frac{\text{d}}{\text{d}t}=\frac{\partial }{\partial t_{0}}+M^{2}\log M\frac{\partial }{\partial t_{1}}+M^{2}\frac{\partial }{\partial t_{2}}+o(M^{2}).$$

Hence $\text{d}\bar{\zeta }/\text{d}t=W=W_{0}+M^{2}\log MW_{1}+M^{2}W_{2}+o(M^{2})$, so that $W_{0}={\bar{\zeta }}_{,0}$, $W_{1}={\bar{\zeta }}_{,1}$, $W_{2}={\bar{\zeta }}_{,2}$, where we write $\zeta_{,i}=\partial \zeta /\partial t_{i}$. We can now write pressure and density using the full time dependence by expanding (2.3) and (2.4) written in complex notation

3.7 $$\begin{array}{rl} p_{0} & \;=-\frac{1}{2}(|w_{0}{|}^{2}+F_{0,0}+{\bar{F}}_{0,0}), \end{array}$$

3.8 $$\begin{array}{rl} p_{1} & \;=-\frac{1}{2}(F_{0,1}+{\bar{F}}_{0,1}), \end{array}$$

3.9 $$\begin{array}{rl} p_{2} & \;=-\frac{1}{2}(F_{0,2}+{\bar{F}}_{0,2})+\frac{1}{8}{\left(|w_{0}{|}^{2}+F_{0,0}+{\bar{F}}_{0,0}\right)}^{2}\\ & \;\;-\frac{1}{2}(w_{0}{\bar{w}}_{2}+{\bar{w}}_{0}w_{2}+F_{2,0}+{\bar{F}}_{2,0}) \end{array}$$

3.10 $$\begin{array}{rl} \mathrm{and}\;\rho_{2} & \;=-\frac{1}{2}(|w_{0}{|}^{2}+F_{0,0}+{\bar{F}}_{0,0}). \end{array}$$

There is a dynamically irrelevant component $p_{-2}={\left(\gamma M^{2}\right)}^{-1}$ that can be ignored, while $\rho_{0}=1$.

### (b) Local solution for the $O(M^{2})$ potential

We now consider the solution near the vortex located at z=ζ. The following expansions provide the terms needed to compute the equations of motion, writing ϵ=z−ζ and e=|ϵ|:

3.11 $$\begin{array}{rl} F_{0} & \;=-\text{i}\kappa \log\epsilon +f_{0}+f_{1}\epsilon +\frac{1}{2}f_{2}\epsilon^{2}+\frac{1}{3}f_{3}\epsilon^{3}+o(e^{3}), \end{array}$$

3.12 $$\begin{array}{rl} F_{0,0} & \;=\frac{\text{i}\kappa \zeta_{,0}}{\epsilon }+f_{0,0}-f_{1}\zeta_{,0}+(f_{1,0}-f_{2}\zeta_{,0})\epsilon +o(e), \end{array}$$

3.13 $$\begin{array}{rl} F_{0,00} & \;=\text{i}\kappa \left[\frac{\zeta_{,00}}{\epsilon }+\frac{\zeta_{,0}^{2}}{\epsilon^{2}}\right]+f_{0,00}-{\left(f_{1}\zeta_{,0}\right)}_{,0}-(f_{1,0}-f_{2}\zeta_{,0})\zeta_{,0}+o(1), \end{array}$$

3.14 $$\begin{array}{rl} J & \;=\text{i}\kappa \left[\zeta_{,00}\log\epsilon -\frac{\zeta_{,0}^{2}}{\epsilon }\right]+J_{0}+[f_{0,00}-{\left(f_{1}\zeta_{,0}\right)}_{,0}-(f_{1,0}-f_{2}\zeta_{,0})\zeta_{,0}]\epsilon +o(e), \end{array}$$

3.15 $$\begin{array}{rl} w_{0} & \;=-\frac{\text{i}\kappa }{\epsilon }+f_{1}+f_{2}\epsilon +f_{3}\epsilon^{2}+o(e^{2}) \end{array}$$

3.16 $$\begin{array}{rl} \mathrm{and}\;I & \;=\frac{\kappa^{2}}{\epsilon }-2\text{i}\kappa f_{1}\log\epsilon +I_{0}+(-2\text{i}\kappa f_{2}+f_{1}^{2})\epsilon +(-\text{i}\kappa f_{3}+f_{1}f_{2})\epsilon^{2}+o(e^{2}). \end{array}$$

We have expressed the coefficients of $F_{0}$ in the above form for later convenience. The coefficients $f_{0}$, $f_{1},\ldots$ depend on time in general. While the coefficient $f_{0}$ appears to have no dynamical meaning, it is different from one vortex to another and hence is kept.

The first step is to ensure that the velocity field obtained from $F_{2}$ in (3.4) is single-valued. While $F_{0}$ has a multi-valued logarithmic term near ζ, the resulting complex velocity, $w_{0}$, is single-valued. However, $F_{2}$ contains logarithmic terms of the form $\log\bar{\epsilon }$ multiplied by functions of *z*. The resulting velocity is not single-valued, but becomes single-valued when adding a homogeneous solution with the same *z*-dependence multiplied by log⁡ϵ. This corresponds to including the following contribution in G(z):

3.17 $$l(z)\log\left(z-\zeta \right)=\left[\frac{1}{4}(z-Z_{0})(-\text{i}\kappa {\bar{\zeta }}_{,00})+\frac{1}{2}F_{0,0}(z)(\text{i}\kappa )+\frac{1}{4}w_{0}(z)(2\text{i}\kappa {\bar{f}}_{1})\right]\log\epsilon .$$

The square bracket defines the function l(z). The effect of (3.17) in the expansion of $F_{2}$ near a vortex is to replace log⁡ϵ terms by $\log e^{2}$.

We then include two homogeneous terms in G(z)

3.18 $$\frac{\mu }{z-\zeta }+\xi \log\left(z-\zeta \right).$$

The coefficients μ and ξ allow us to remove unacceptable singularities in $F_{2}$ near ϵ=0. Since $F_{0}$ has logarithmic singularities, $F_{2}$ cannot have singularities of higher order, or else the expansion would be disordered near ϵ=0. Logarithmic singularities in $F_{2}$ are removed by requiring the vorticity to be entirely at O(1). Unlike [8] we do not require a term in $\epsilon^{-2}$.

Finally, the local expansion of G(z) also contains terms from expanding the counterparts of the terms inside the square bracket in (3.4) due to other vortices and to other homogeneous contributions to G(z). From the forms of $F_{0,0}$ and of I(z), the former lead to terms of $O(e^{-1})$ as well as analytic terms. The latter are denoted by K(z). The result is a contribution $g_{-1}\epsilon^{-1}+g_{0}+g_{1}\epsilon +O(e^{2})$ near the vortex. (These contributions are calculated explicitly for the two-vortex case in §4.)

We can now write down the expansion for $F_{2}$ needed to remove singular terms:

3.19 $$\begin{array}{rl} F_{2} & \;=-\frac{\text{i}\kappa^{3}}{4|\epsilon {|}^{2}}+\frac{\kappa^{2}\log\;e^{2}}{4\epsilon }(-2\zeta_{,0}+2{\bar{f}}_{1})+\frac{1}{4\epsilon }(2\text{i}\kappa {\bar{f}}_{0}\zeta_{,0}\\ & \;\;-\text{i}\kappa \bar{I_{0}}+4\mu +4g_{-1})+\frac{\kappa^{2}}{4\bar{\epsilon }}f_{1}+\frac{\text{i}\kappa }{4\bar{\epsilon }}(\zeta -Z_{0})\bar{\zeta_{,0}^{2}}\\ & \;\;+\frac{1}{4}[2\text{i}\kappa (f_{0,0}-f_{1}\zeta_{,0})+2\text{i}\kappa |f_{1}{|}^{2}-\text{i}\kappa (\zeta -Z_{0}){\bar{\zeta }}_{,00}]\log e^{2}+\xi \log\epsilon +O(1). \end{array}$$

The term in $\epsilon^{-2}$ is purely imaginary and hence can be ignored, while the term in $\epsilon^{-1}\log e^{2}$ cancels from the O(1) result, which will be rederived in the current framework and notation below. Since $F_{2}$ only enters the solution via its real part, removing the singular terms in its real part leads to the following conditions:

3.20 $$2\text{i}\kappa {\bar{f}}_{0}\zeta_{,0}-\text{i}\kappa (\bar{\zeta -Z_{0}})\zeta_{,0}^{2}-\text{i}\kappa {\bar{I}}_{0}+4\mu +\kappa^{2}{\bar{f}}_{1}+4g_{-1}=0$$

and

3.21 $$2\text{i}\kappa (f_{0,0}-f_{1}\zeta_{,0})-2\text{i}\kappa ({\bar{f}}_{0,0}-{\bar{f}}_{1}{\bar{\zeta }}_{,0})+4\xi -\text{i}\kappa (\zeta -Z_{0}){\bar{\zeta }}_{,00}+\text{i}\kappa (\bar{\zeta -Z_{0}})\zeta_{,00}=0.$$

### (c) Conservation of momentum

We now return to the conservation of momentum, viewing it as a matching problem for an expansion in *M*, with *e* serving as the independent variable. We define *Q* as the left-hand side of (1.1) and express it as an expansion in the inner variable, *s*, and define *q* as the right-hand side and express it as an expansion in the vortex variable, *e*. We expand the time-derivative of (2.8) in the inner variable, using $W=W_{0}+M^{2}\log MW_{1}+M^{2}W_{2}+\cdots$ and $\text{d}/\text{d}t=\partial_{{\;}_{,}0}+M^{2}\log M\partial_{{\;}_{,}1}+M^{2}\partial_{,2}+\cdots$, giving

3.22 $$\begin{array}{rl} Q & \;=\pi [{\bar{W}}_{0,0}+M^{2}\log M({\bar{W}}_{0,1}+{\bar{W}}_{1,0})+M^{2}({\bar{W}}_{0,2}+{\bar{W}}_{2,0})+o(M^{2})]\\ & \;\;\times [M^{2}s^{2}-\kappa^{2}M^{2}\log s+M^{2}C+O(M^{2}s^{-2}))]+O(M^{2}), \end{array}$$

where the final $O(M^{2})$ term includes an as yet unknown dependence on *s*. In anticipation of using Van Dyke’s rule (e.g. [16]), we employ the notation $Q^{\left(n,.\right)}$ to denote the *n*-term truncation of the function *Q* and $Q^{\left(n,m\right)}$ to denote its subsequent truncation to *m* terms when rewritten in the outer variable. Then $Q_{0}=Q^{\left(0,.\right)}=0$ since there are no O(1) terms in (3.22). Expanding the right-hand side of (1.1) leads to the exact result

3.23 $$\begin{array}{rl} q_{0} & \;=\text{i}\oint_{C}p_{0}\;\text{d}z-\frac{\text{i}}{2}\oint_{C}{\bar{w}}_{0}[({\bar{w}}_{0}-{\bar{W}}_{0})\;\text{d}\bar{z}-(w_{0}-W_{0})\;\text{d}z]\\ & \;=\pi \text{i}\kappa [{\bar{W}}_{0}-2{\bar{f}}_{1}+\zeta_{,0}]+\pi [{\bar{f}}_{1,0}-{\bar{f}}_{2}({\bar{\zeta }}_{,0}-W_{0})]\;e^{2}. \end{array}$$

The (0,0) term in Van Dyke’s rule is $Q^{\left(0,0\right)}=q^{\left(0,0\right)}$, so that

3.24 $$0=\pi \text{i}\kappa [{\bar{W}}_{0}-2{\bar{f}}_{1}+\zeta_{,0}].$$

Using $\zeta_{,0}={\bar{W}}_{0}$ leads to

3.25 $${\bar{\zeta }}_{,0}=W_{0}=f_{1},$$

i.e. the incompressible result expressed in the current notation.

We should now group the $O(M^{2}\log M)$ and $O(M^{2})$ terms together to continue with Van Dyke’s rule. We should also compute $Q^{\left(2,.\right)}$. We shall avoid doing this, and instead carry out the matching informally. This approach works, but to be safe we will revisit the formal matching in the electronic supplementary material. In the vortex region, the right-hand side of (1.1) gives the $O(M^{2}\log M)$ contribution

3.26 $$\begin{array}{rl} q_{1} & \;=\text{i}\oint_{C}p_{1}\;\text{d}z-\frac{\text{i}}{2}\oint_{C}{\bar{w}}_{0}[-{\bar{W}}_{1}\;\text{d}\bar{z}+W_{1}\;\text{d}z]\\ & \;=\pi \text{i}\kappa [{\bar{W}}_{1}+\zeta_{,1}]+\pi [{\bar{f}}_{1,1}-{\bar{f}}_{2}({\bar{\zeta }}_{,1}-W_{1})]\;e^{2}. \end{array}$$

These two terms correspond to the terms

3.27 $$\pi M^{2}\log M[{\bar{W}}_{0,0}+({\bar{W}}_{0,1}+{\bar{W}}_{1,0})\;e^{2}]$$

in (3.22). The constant term gives the evolution equation on the timescale $t_{1}$ as

3.28 $$\zeta_{,1}=-\frac{\text{i}\kappa }{2}{\bar{W}}_{0,0}=-\frac{\text{i}\kappa }{2}\zeta_{,00}.$$

At this point, the terms at $O(e^{2})$ do not match. This is because the matching requires further terms in *Q*, as discussed in the electronic supplementary material.

The $O(M^{2})$ contribution is

3.29 $$\begin{array}{rl} q_{2} & \;=\text{i}\oint_{C}p_{2}\;\text{d}z-\frac{\text{i}}{2}\oint_{C}\rho_{2}{\bar{w}}_{0}[(W_{0}-{\bar{W}}_{0})\;\text{d}\bar{z}-(w_{0}-W_{0})\;\text{d}z]\\ & \;\;-\frac{\text{i}}{2}\oint_{C}{\bar{w}}_{2}[({\bar{w}}_{0}-{\bar{W}}_{0})\;\text{d}\bar{z}-(w_{0}-W_{0})\;\text{d}z]\\ & \;\;-\frac{\text{i}}{2}\oint_{C}{\bar{w}}_{0}[({\bar{w}}_{2}-{\bar{W}}_{2})\;\text{d}\bar{z}-(w_{2}-W_{2})\;\text{d}z]. \end{array}$$

To obtain this, we need to consider further terms in the local expansion of $F_{2}$. Using the conditions (3.20) and (3.21), we find

3.30 $$F_{2}≗A+B\frac{\epsilon }{\bar{\epsilon }}+D\epsilon \log\;e^{2}+H\epsilon +L\frac{\epsilon^{2}}{\bar{\epsilon }}+o(e),$$

where the relation ≗ means that the equality ignores purely imaginary terms. The coefficients needed are

3.31 $$\begin{array}{rl} D & \;=\frac{\text{i}\kappa }{4}[-{\bar{\zeta }}_{,00}+2(f_{1,0}-f_{2}\zeta_{,0})+2{\bar{f}}_{1}f_{2}]=\frac{\text{i}\kappa }{4}f_{1,0}, \end{array}$$

3.32 $$\begin{array}{rl} H & \;=\frac{1}{4}[{\bar{J}}_{0}+2{\bar{f}}_{0}(f_{1,0}-f_{2}\zeta_{,0})+f_{2}{\bar{I}}_{0}+2f_{1}({\bar{f}}_{0,0}-{\bar{f}}_{1}{\bar{\zeta }}_{,0})+{\bar{f}}_{1}(-2\text{i}\kappa f_{2}+f_{1}^{2})]\\ & \;\;+\frac{1}{4}(\bar{\zeta }-\bar{Z_{0}})[f_{0,00}-{\left(f_{1}\zeta_{,0}\right)}_{,0}-(f_{1,0}-f_{2}\zeta_{,0})\zeta_{,0}]+g_{1}. \end{array}$$

The expansion (3.30) leads to

3.33 $$w_{2}=\frac{B}{\bar{\epsilon }}-\bar{B}\frac{\bar{\epsilon }}{\epsilon^{2}}+D(\log e^{2}+1)+\bar{D}\frac{\bar{\epsilon }}{\epsilon }+H+2L\frac{\epsilon }{\bar{\epsilon }}-\bar{L}\frac{\epsilon^{2}}{{\bar{\epsilon }}^{2}}+o(1)$$

and

3.34 $$F_{2,0}≗B\left({\bar{\zeta }}_{,0}\frac{\epsilon }{{\bar{\epsilon }}^{2}}-\frac{\zeta_{,0}}{\bar{\epsilon }}\right)+o(e^{-1}).$$

Substituting into (3.29) and computing the integrals leads to extensive cancellation, yielding

3.35 $$q_{2}=-\frac{\pi }{2}\kappa^{2}{\bar{f}}_{1,0}+\pi \text{i}\kappa \zeta_{,2}+\pi \text{i}\kappa {\bar{W}}_{2}-2\pi \text{i}\kappa \bar{H}-2\pi \text{i}\kappa \bar{D}(1+\log e^{2})+o(1).$$

We see from (3.22) that the log⁡e term in $q_{2}$ cancels the log⁡e term at O$\left(M^{2}\right)$ in *Q*. Recalling that $\zeta_{,2}={\bar{W}}_{2}$ gives the equation for the slow evolution of ζ as

3.36 $$\zeta_{,2}=-\frac{\text{i}{\bar{W}}_{0,0}}{2\kappa }C-\frac{\text{i}\kappa }{4}{\bar{f}}_{1,0}+\bar{D}+\bar{H}=-\frac{\text{i}\kappa }{2}{\bar{f}}_{1,0}\left(1+\frac{C}{\kappa^{2}}\right)+\bar{H}.$$

It is useful to check the behaviour of a single point vortex. The incompressible complex potential is $F_{0}=-\text{i}\kappa \log\left(z-\zeta \right)$. The point vortex does not move at O(1). Hence $\zeta_{,1}=0$ and

3.37 $$F_{2}=\frac{1}{4}w_{0}\bar{I(z)}+G(z)=\frac{1}{4}\left(-\frac{\text{i}\kappa }{z-\zeta }\right)\left[\frac{\kappa^{2}}{\bar{z}-\bar{\zeta }}-\frac{\kappa^{2}}{{\bar{z}}_{I}-\bar{\zeta }}\right]+\frac{\mu }{z-\zeta }.$$

The arbitrariness of $z_{I}$ is irrelevant, as it is cancelled by $\mu_{1}$ when removing the simple pole in $F_{2}$. The leading-order term is purely imaginary so it can be ignored. Hence the $O(M^{2})$ velocity of a single point vanishes, a necessary feature for this model.

### (d) Global solution

The results above are applicable near every vortex, because neither the vortex circulation nor the vortex location has a preferred value. We can now assemble a global solution that is valid everywhere in the vortical region. The $O(M^{2})$ potential is given by the sum of the inhomogeneous part (3.4) and of a homogeneous part. The homogeneous part takes the form

3.38 $$G(z)=\sum_{m}\left(\frac{\mu_{m}}{z-\zeta_{m}}+[\xi_{m}+l_{m}(z)]\log\left(z-\zeta_{m}\right)\right)+K(z),$$

and includes contributions from each vortex of the form (3.17) and (3.18), while K(z) includes possible further terms (e.g. to satisfy boundary conditions or to set the circulation around objects in the flow). We now consider the expansion near vortex *n* of the sum in (3.38) omitting term *n*, writing the rest of the sum as $g_{-1}\epsilon_{n}^{-1}+g_{0}+g_{1}\epsilon_{n}+O(e_{n}^{2})$, with $\epsilon_{n}=z-\zeta_{n}$ and $e_{n}=|\epsilon_{n}|$. The calculations above show that $g_{0}$ is not needed. We find, for vortex *n*,

3.39 $$g_{-1}=\frac{1}{2}\kappa_{n}\sum_{m}{\;}^{\prime }\kappa_{m}(\bar{f_{1}^{\left(m\right)}}-\zeta_{n,0})\log\zeta_{mn}=\frac{1}{2}\kappa_{n}\sum_{m}{\;}^{\prime }\kappa_{m}(\zeta_{m,0}-\zeta_{n,0})\log\zeta_{mn}$$

and

3.40 $$g_{1}=\sum_{m}{\;}^{\prime }\left(-\frac{\mu_{m}}{\zeta_{mn}^{2}}+\frac{\xi_{m}+l_{0}^{\left(mn\right)}}{\zeta_{mn}}+l_{1}^{\left(mn\right)}\log\zeta_{mn}\right)+K^{\prime }(\zeta_{n}),$$

where $\zeta_{mn}=\zeta_{n}-\zeta_{m}$ and the prime in the summation indicates that term *n* is omitted (the derivation is given in the electronic supplementary material).

## 4. Two vortices in the plane

We consider the simplest situation consisting of two point vortices in the infinite plane. In cases such as this, there are no other contributions to the potential $F_{0}$ beyond the vortices, which means that K(z)=0 in (3.38). While some simple geometries can also be solved using the method of images and could hence be considered as consisting of a finite number of vortices, the dynamics of the actual and image vortices are different: the latter are not physical so that their motion is set by boundary conditions rather than matching.

Results for the leading-order potential, complex velocity, *I* and *J* are equally simple for *N* vortices; we will take N=2 after presenting general results. We have

4.1 $$F_{0}=\sum_{n=1}^{N}\frac{\Gamma_{n}}{2\pi \text{i}}\log\left(z-\zeta_{n}\right)\;\mathrm{and}\;w_{0}=\sum_{n=1}^{N}\frac{\Gamma_{n}}{2\pi \text{i}}\frac{1}{z-\zeta_{n}}.$$

The decay properties of w(z) for large |z| mean that we can take $z_{I}=\infty$. It is known that the incompressible two- and three-vortex cases are integrable. The system has four real conserved quantities, two of which combine to give the complex vortex momentum $\sum_{n=1}^{N}\Gamma_{n}\zeta_{n}$. Conservation of this quantity means that integral J(z) is convergent at infinity, so that we can take $z_{J}=0$. To calculate the integrals *I* and *J*, we use the primitives

4.2 $$\int^{z}\sum_{m}{\left[-\frac{\text{i}\kappa_{m}}{z-\zeta_{m}}\right]}^{2}\;\text{d}z=\sum_{m}\frac{\kappa_{m}^{2}}{z-\zeta_{m}}+\sum_{m,n}{\;}^{\prime }\frac{\kappa_{m}\kappa_{n}}{\zeta_{nm}}\log\frac{z-\zeta_{n}}{z-\zeta_{m}}$$

and

4.3 $$\int^{z}\sum_{m}\text{i}\kappa_{m}\left[\frac{\zeta_{m,00}}{z-\zeta_{m}}+\frac{\zeta_{m,0}^{2}}{{\left(z-\zeta_{m}\right)}^{2}}\right]\;\text{d}z=\sum_{m}\text{i}\kappa_{m}\left[\zeta_{m,00}\log\left(z-\zeta_{m}\right)-\frac{\zeta_{m,0}^{2}}{z-\zeta_{m}}\right],$$

where the primed sums indicate m≠n.

For the two-vortex case, the total circulation is $\Gamma_{\infty }=\Gamma_{1}+\Gamma_{2}$, so there are two different cases, corresponding to $\Gamma_{\infty }=0$ and $\Gamma_{\infty }\neq 0$. In the latter case, the conservation laws show that the vortices must stay in a bounded area of the plane. The former case corresponds to a co-propagating dipole pair in the incompressible limit. Expressions for $f_{0}$, $f_{1}$, $J_{0}$ and $g_{-1}$ for the two vortices are given in the electronic supplementary material. The relation (3.21) gives $\xi_{1}=\xi_{2}=0$ if $Z_{0}$ is taken to be on the line joining $\zeta_{1}$ and $\zeta_{2}$, although the final result for the motion of the vortices is independent of $Z_{0}$.

In the co-propagating case, we can take $\kappa =\kappa_{1}=-\kappa_{2}$. Then $\zeta_{21}$ and $|\zeta_{1}{|}^{2}-|\zeta_{2}{|}^{2}$ are independent of $t_{0}$. Without loss of generality, we take the positions of the vortices at t=0 to be $\pm \text{i}a_{0}$ with $a_{0}$ real, yielding

4.4 $$\zeta_{1}=\text{i}a+\frac{\kappa t}{2a}\;\mathrm{and}\;\zeta_{2}=-\text{i}a+\frac{\kappa t}{2a},$$

with $a=a(t_{1},t_{2})$ and $a(0,0)=a_{0}$. Since $\zeta_{1,00}=\zeta_{2,00}=0$, we find from (3.28) that $\zeta_{1}$ and $\zeta_{2}$ do not depend on $t_{1}$, so that $a=a(t_{2})$. Since $f_{1,0}=0$, D=0, and since ${\left(\zeta_{1}-\zeta_{2}\right)}_{,0}=0$, we have $g_{-1}=0$. Calculations (see the electronic supplementary material) lead to $H_{1}=0$, with the $Z_{0}$ terms cancelling. Substituting into (3.36), along with $f_{1,0}=0$, means that ζ does not change with $t_{2}$. Hence we recover the result of L06 and [8]: the translation speed of the co-propagating vortex pair does not change at $O(M^{2})$. The current procedure is of course lengthier than that needed to obtain this result in a co-moving frame in which the pair is at rest, but we can now address the fundamentally unsteady co-rotating pair.

For the co-rotating case, $\kappa =\kappa_{1}=\kappa_{2}$, so that $\zeta_{1}+\zeta_{2}$ is independent of $t_{0}$. Without loss of generality, we take the positions of the vortices at t=0 to be $\pm a_{0}$ with $a_{0}$ real, yielding

4.5 $$\zeta_{1}=a{\text{e}}^{\text{i}\varphi }\;\mathrm{and}\;\zeta_{2}=-a{\text{e}}^{\text{i}\varphi }$$

with $\varphi =\theta +\kappa t/(2a^{2})=\theta +\omega t$. Here, *a* and θ are functions of $t_{1}$ and $t_{2}$ with $a(0,0)=a_{0}$ and θ(0,0)=0. Since $\zeta_{1,00}=-\omega^{2}\zeta_{1}$, we find from (3.28) that

4.6 $$a_{,1}=0\;\mathrm{and}\;\theta_{,1}=\omega_{1}=\frac{\kappa \omega^{2}}{2}=\frac{\kappa^{3}}{8a^{4}}.$$

The radius is only a function of $t_{2}$, while the rotation rate varies with $t_{1}$. Since the $\theta_{,1}$ term has the same sign as the O(1) rotation rate, it leads to a slowing down of the rotational motion when multiplied by $M^{2}\log M$, which is negative since $0<M^{2}\ll 1$. Fairly extensive algebra (see the electronic supplementary material) gives

4.7 $$H_{1}=-\frac{\text{i}\kappa^{3}}{16a^{3}}{\text{e}}^{-\text{i}\varphi }(1-\log4a^{2})$$

and

4.8 $$\begin{array}{rl} \zeta_{1,2} & \;=-\frac{\text{i}\kappa }{2}{\bar{f}}_{1,0}^{\left(1\right)}\left(1+\frac{C}{\kappa^{2}}\right)+{\bar{H}}_{1}=\frac{\text{i}\kappa^{3}}{8a^{3}}{\text{e}}^{\text{i}\varphi }\left(1+\frac{C}{\kappa^{2}}\right)+{\bar{H}}_{1}\\ & \;=\frac{\text{i}\kappa^{3}}{8a^{4}}\zeta \left(\frac{3}{2}+\frac{C}{\kappa^{2}}-\log2a\right). \end{array}$$

Here, the constant *C* is given in (2.10). Once again there is a correction to the rotation speed

4.9 $$\theta_{,2}=\omega_{2}=\frac{\kappa^{3}}{8a^{4}}\left(\frac{3}{2}+\frac{C}{\kappa^{2}}-\log2a\right).$$

In dimensional form, we can combine the frequencies to obtain

4.10 $$\omega^{*}=\frac{\kappa_{*}}{2a_{*}}\left[1+\frac{\kappa_{*}^{2}}{4c_{0}^{2}a_{*}^{2}}\left(-\log\left(\frac{2a^{*}\Gamma }{c_{0}L^{2}}\right)+\frac{3}{2}+\frac{C\Gamma^{2}}{\kappa_{*}^{2}}\right)\right],$$

where stars represent dimensional quantities. The circulation scale is Γ=LV. There is no unique choice of scalings, but the simplest choice is probably $\Gamma =2\kappa_{*}$ and $L=2a_{*}$, so that Γ is the total circulation divided by 2π and *L* is the distance between vortices. Then

4.11 $$\omega^{*}=\frac{\kappa_{*}}{2a_{*}}\left[1+\frac{\kappa_{*}^{2}}{4c_{0}^{2}a_{*}^{2}}\left(-\log\left(\frac{\kappa_{*}}{c_{0}a_{*}}\right)+\frac{3}{2}+4C\right)\right].$$

For the general two-vortex case, the vortices rotate about their centre of vorticity at O(1). Write $\zeta_{1}=a{\text{e}}^{\text{i}\varphi }$ and $\zeta_{2}=-b{\text{e}}^{\text{i}\varphi }$ with $a\kappa_{1}=b\kappa_{2}$ so that the centre of vorticity is at the origin with $Z_{0}=0$. Then

4.12 $$\omega =\frac{\kappa_{1}}{b(a+b)}=\frac{\kappa_{2}}{a(a+b)}.$$

The $O(M^{2}\log M)$ equations become

4.13 $$\zeta_{1,1}=-\zeta_{2,1}=\frac{\text{i}\kappa_{1}\kappa_{2}\omega }{2{\bar{\zeta }}_{21}}.$$

The velocities of the vortices are the same, but their angular velocities differ, so that as they move their trajectories will no longer be circles. The centre of the vorticity $\zeta_{c}=(\kappa_{1}\zeta_{1}+\kappa_{2}\zeta_{2})/(\kappa_{1}+\kappa_{2})$ moves slowly with the $O(M^{2}\log M)$ velocity

4.14 $$\zeta_{c,1}=\frac{\text{i}\kappa_{1}\kappa_{2}(\kappa_{1}-\kappa_{2})\omega }{2{\bar{\zeta }}_{21}(\kappa_{1}+\kappa_{2})}.$$

The $O(M^{2})$ motion is

4.15 $$\begin{array}{rl} & \zeta_{1,2}=\frac{\text{i}\kappa_{1}\kappa_{2}(\kappa_{1}+\kappa_{2})}{4|\zeta_{21}{|}^{2}{\bar{\zeta }}_{21}}\left(3+\frac{2C_{1}}{\kappa_{1}^{2}}-\log|\zeta_{21}{|}^{2}\right)\\ \mathrm{and}\; & \zeta_{2,2}=\frac{\text{i}\kappa_{1}\kappa_{2}(\kappa_{1}+\kappa_{2})}{4|\zeta_{12}{|}^{2}{\bar{\zeta }}_{12}}\left(3+\frac{2C_{2}}{\kappa_{2}^{2}}-\log|\zeta_{12}{|}^{2}\right). \end{array}$$

This slow motion can be decomposed into rotation about a slowly moving centre with the $O(M^{2})$ correction

4.16 $$\zeta_{c,2}=\frac{\text{i}\kappa_{1}\kappa_{2}(\kappa_{1}-\kappa_{2})}{4|\zeta_{21}{|}^{2}{\bar{\zeta }}_{21}}\left(3+\frac{2(C_{1}\kappa_{2}-C_{2}\kappa_{1})}{\kappa_{1}\kappa_{2}(\kappa_{1}-\kappa_{2})}-\log|\zeta_{21}{|}^{2}\right).$$

This shows that the relative equilibrium of the co-rotating vortices evolves slowly in time due to the weak compressibility effects, although in the symmetric case, it is only the rotation rate that changes.

## 5. Outer region

As pointed out by L06, at distances of $O(M^{-1}$) from the vortices, there is a region in which the dynamics are wave-like. We define far-field upper-case variables by $X=M\hat{x}$. The Blokhintsev equation becomes

5.1 $$\begin{array}{rl} \frac{\partial^{2}\Phi }{\partial X_{i}^{2}}-\frac{\partial^{2}\Phi }{\partial t^{2}} & \;=(\gamma -1)M^{2}\left(\frac{\partial \Phi }{\partial t}+\frac{1}{2}M^{2}\frac{\partial \Phi }{\partial X_{j}}\frac{\partial \hat{\Phi }}{\partial X_{j}}\right)\frac{\partial^{2}\Phi }{\partial X_{i}^{2}}\\ & \;\;+2M^{2}\frac{\partial \Phi }{\partial X_{i}}\frac{\partial^{2}\Phi }{\partial X_{i}\partial t}+M^{4}\frac{\partial \Phi }{\partial X_{i}}\frac{\partial \phi }{\partial X_{j}}\frac{\partial^{2}\Phi }{\partial X_{i}\partial X_{j}}. \end{array}$$

One can obtain expressions for the pressure and density analogous to (2.3) and (2.4).

The far-field limit of the O(1) potential in the vortical region is

5.2 $$F_{0}=-\text{i}\kappa_{\infty }\log z+g_{0}+\frac{g_{1}}{z}+\frac{g_{2}}{z^{2}}+O(|z{|}^{-3}),$$

where the total circulation is $2\pi \kappa_{\infty }$ and the $g_{i}(t)$ are functions of time alone. The incompressible dynamics of the vortex region imply that $\kappa_{\infty }$, and $g_{1}$, which is related to the vortex impulse *I* [2], are independent of time. We set $g_{0}=0$ as the global constant of integration for $F_{0}$. Rewritten in the outer variable, we have

5.3 $$F_{0}=-\text{i}\kappa_{\infty }\log MZ+M\frac{g_{1}}{Z}+M^{2}\frac{g_{2}}{Z^{2}}+O(M^{3}|Z{|}^{-3}).$$

From (5.1), $\Phi_{0}$ is a harmonic function, which must be $\kappa_{\infty }\theta$, so that the real part matches. Since the higher terms in (5.1) are multiplied by powers of $M^{2}$, $\Phi_{1}$ is again a potential function, and must be $\text{Re}(g_{2}Z^{-2})$. This result requires checking the behaviour of $F_{2}$ for large |z|. We have $F_{0,0}=O(|z{|}^{-3})$, $J=O(|z{|}^{-2})$, $w_{0}=O(|z{|}^{-1})$, $I=O(|z{|}^{-1})$, so that the inhomogeneous terms in (3.4) are $O(z^{-1})$, while the sum of the separate l(z) terms is $O(|z{|}^{-1}\log|z|)$ in the infinite-plane case in which $\sum_{n}\zeta_{n,0}=0$. Finally, the homogeneous terms from (3.18) are $O(|z{|}^{-1})$ since $\sum \xi_{n}=0$. Hence the terms in $F_{2}$ do not enter the matching at $O(M^{2}$).

The general question of compressible far-field waves relates to the domain of aeroacoustics. Excellent reviews of aeroacoustics are given e.g. in [12,17]. The use of MAE in solving aeroacoustic problems is reviewed in [13]. For the general case, we can use these results. We hence summarize the necessary results following [12]: wavelike solutions with dipole $R^{-2}\cos\left(2\theta -\chi \right)$ behaviour near the origin are given by a synthesis of monochromatic solutions of the form

5.4 $$\text{Re}[H_{2}^{\left(0\right)}(R){\text{e}}^{\pm 2\text{i}\theta }{\text{e}}^{\text{i}\omega t}]$$

that satisfy the radiation condition. Here, $H_{2}^{\left(0\right)}$ is the Hankel function of the second kind. These solutions are superposed, with amplitude coefficients A(ω) that can be related to $g_{2}$. One obtains quadrupole radiation of flow-generated sound, as expected in a situation with no boundaries or mass sources.

For the two cases considered in §3, we can follow previous authors directly. Acoustic emission from the co-rotating vortex pair was first examined by [18], who found a change of rotation frequency equivalent to that in §3. The velocity of the co-propagating pair is found to be independent of $t_{2}$, $t_{1}$ and $t_{0}$, so the analysis of L06, who considered a steady dipole in a moving reference frame, is equivalent to the current one. L06 shows by using a coordinate system moving with the vortex that the O(M) solution, a propagating dipole, is efficiently represented in the coordinate system $\left({\left(1-M^{2}\right)}^{1/2}X,Y\right)$. This is equivalent to a different way of writing an asymptotic solution that gives the same result to the order obtained.

## 6. Conclusion

We have obtained equations of motion for small-Mach-number compressible point vortices in the plane, in which compressibility manifests itself as an evolution over slow time scales of $O(M^{2}\log M)$ and $O(M^{2})$. The first correction (3.28) is quite simple and vanishes for steady configurations. The second correction is more involved.

We have examined the corrections to $O(M^{2})$ for the simplest case of vortex pairs. We recover the known result for the co-propagating pair that the velocity is unchanged at $O(M^{2})$. The symmetric co-rotating vortex pair exhibits a change of angular velocity. For the general two-vortex case, however, the centre of rotation and radius of the orbit evolve slowly, while the motion of each vortex is instantaneously perpendicular to the line of centres and the motion remains circular on the O(1) time scale.

The solution in the far-field region with spatial scale $M^{-1}L$, corresponding to the wavelength of the emitted sound, can be obtained by matching, following previous work. If the vortical flow is steady, the response is a dipole moving with the speed of the centre of vorticity, as in L06. If the vortical flow is unsteady, an expression for the quadrupole radiation is obtained in terms of the quadrupole moment $g_{2}(t)$ (presumably this could be applied to the calculation of wave radiation by chaotic point vortex evolution as in [19,20]). Following previous work, quantities such as the power radiated to infinity could be obtained.

The back-reaction of the wave field is deliberately ignored here, as is usual in aeroacoustics. This means that while radiation is present in the current formulation, the coupling of the flow in the vortical region to the far field only appears at an order higher than $M^{2}$. In the geophysical context, the corresponding effect of gravity wave radiation on vortex dynamics has been examined [21] (see also [22] for a related discussion for scattering). Here, this would require a calculation to $O(M^{4})$, most likely with logarithmic terms.

A list of interesting extensions comes to mind: efforts at simplifying the equations further in special cases; possible efficient solution techniques; whether any of the other known equilibria of point vortices survive to $O(M^{2})$; the effects of more complicated boundaries and whether a better model for the core regions is warranted. The effect of boundaries is of particular interest, following on from BNEEY. The case of vortices inside and outside a circle is currently being examined: the O(1) solution can be obtained using the method of images, but the corrections require extensive algebra. A further example is the half-plane with a vortex moving around it considered in [23]). Finally, it could be interesting to compare the present results to numerical simulations. These would require very highly resolved aeroacoustic-type calculations with large separations of scales between vortex cores, vortical region and wave field.^1^

## Data Availability

The data are provided in electronic supplementary material [24].

## Footnotes

1 Solving the Gross–Pitaevskii (GP) model, which is almost isomorphic to the irrotational two-dimensional compressible Euler equation under a Madelung transform, might provide one way to proceed numerically, although the details of the vortex cores would be different.
